# Variation of colorectal, breast and prostate cancer screening activity in Switzerland: Influence of insurance, policy and guidelines

**DOI:** 10.1371/journal.pone.0231409

**Published:** 2020-04-16

**Authors:** Agne Ulyte, Wenjia Wei, Holger Dressel, Oliver Gruebner, Viktor von Wyl, Caroline Bähler, Eva Blozik, Beat Brüngger, Matthias Schwenkglenks

**Affiliations:** 1 Department of Epidemiology, Epidemiology, Biostatistics & Prevention Institute, University of Zurich, Zurich, Switzerland; 2 Division of Occupational and Environmental Medicine, Department of Epidemiology, Epidemiology, Biostatistics & Prevention Institute, University of Zurich and University Hospital Zurich, Zurich, Switzerland; 3 Department of Geography, University of Zurich, Zurich, Switzerland; 4 Department of Health Sciences, Helsana Group, Dubendorf, Switzerland; 5 Division of General Practice, University Medical Centre Freiburg, Freiburg, Germany; King’s College London, UNITED KINGDOM

## Abstract

Variation in utilization of healthcare services is influenced by patient, provider and healthcare system characteristics. It could also be related to the evidence supporting their use, as reflected in the availability and strength of recommendations in clinical guidelines. In this study, we analyzed the geographic variation of colorectal, breast and prostate cancer screening utilization in Switzerland and the influence of available guidelines and different modifiers of access. Colonoscopy, mammography and prostate specific antigen (PSA) testing use in eligible population in 2014 was assessed with administrative claims data. We ran a multilevel multivariable logistic regression model and calculated Moran’s I and regional level median odds ratio (MOR) statistics to explore residual geographic variation. In total, an estimated 8.1% of eligible persons received colonoscopy, 22.3% mammography and 31.3% PSA testing. Low deductibles, supplementary health insurance and enrollment in a managed care plan were associated with higher screening utilization. Cantonal breast cancer screening programs were also associated with higher utilization. Spatial clustering was observed in the raw regional utilization of all services, but only for prostate cancer screening in regional residuals of the multilevel model. MOR was highest for prostate cancer screening (1.24) and lowest for colorectal cancer screening (1.16). The reasons for the variation of the prostate cancer screening utilization, not recommended routinely without explicit shared decision-making, could be further investigated by adding provider characteristics and patient preference information. This first cross-comparison of different cancer screening patterns indicates that the strength of recommendations, mediated by specific health policies facilitating screening, may indeed contribute to variation.

## Introduction

Patient, provider and healthcare system characteristics may influence access to healthcare and can be defined in terms of personal, financial and organizational barriers and facilitators [1]. Regional variation of healthcare utilization is one indicator of unequal access [2]. However, such variation may also be related to the characteristics of the service itself–whether it is preference-sensitive, how convincing the evidence is for the service’s effect on health outcomes, and whether it is known and accepted by providers and patients [3]. High quality of the available guidelines and supporting evidence may facilitate the service’s implementation and reduce variation in utilization [4].

Analysis of cancer screening utilization offers an opportunity to explore the impact of evidence-based guidelines on the variation of the utilization of healthcare services. All cancer screening services have broadly the same goal–detection of a symptomless early-stage cancer. Target populations are often defined by sex and age criteria only. However, cancer screening services differ in the available evidence of their impact on health outcomes. For example, colorectal cancer screening is recommended routinely as it has been shown to reduce mortality [5], breast cancer screening is often recommended, but the balance of benefits and harms is debated in Switzerland and worldwide [6,7], and routine screening of prostate cancer is discouraged by Swiss and European guidelines due to lacking evidence [8–10]. Potentially, differences in supporting evidence may lead to different utilization and regional variation patterns.

**Fig 1:**
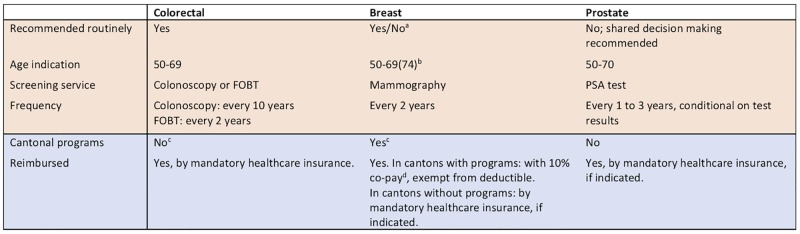
Screening for colorectal, breast and prostate cancer: Recommendations and reimbursement in Switzerland in 2014. FOBT–fecal occult blood test, PSA–prostate specific antigen. ^a^ breast cancer screening is often recommended but the balance of benefits and harms is debated in Switzerland and worldwide. ^b^ recommended age range (50–69 or 50–74) depends on the canton. ^c^ for colorectal cancer screening, programs in two cantons were introduced on July 1, 2014, and were effective from 2015. For breast cancer screening, cantons with a program effective from the beginning of 2014 are depicted in S1 Fig. ^d^ co-pay was covered in cantons of Jura and Wallis in 2014.

Relevant barriers and facilitators of access that should be controlled to observe the effect of guidelines and evidence include regional policies of promotion and reimbursement rules [11]. In Switzerland, colorectal, breast and prostate cancer screening have different reimbursement and promotion policies (Fig 1). Colorectal cancer screening is reimbursed nationally with mandatory health insurance since 2013, and effectively no cantonal programs were running in 2014. Breast cancer screening is reimbursed in cantons with a screening program regardless of the deductible chosen for the mandatory health insurance, with a 10% out-of-pocket copayment, and promoted by personal invitation letters [12]. Prostate cancer screening is not routinely reimbursed, unless indicated after a shared-decision making process [9].

Other aspects of access to care, such as service availability and personal socioeconomic characteristics, could drive variation in cancer screening utilization [13,14]. Similarly, the publication of trial results and guidelines has been shown to influence utilization rates [15]. However, the effects of modifiers of access and the evidence for screening services are rarely investigated in combination. In Switzerland few studies have investigated the geographic variation in breast cancer screening utilization either at the federal [16] or regional levels [17,18] and only one study has explored the variation of colorectal cancer screening utilization across primary care practices [19]. Therefore, evidence is lacking about how utilization and its regional variation compare across multiple cancer screening services.

We aimed to assess the influence of different modifiers of access and available evidence-based guidelines on the utilization of cancer screening services. Specifically, we aimed to 1) investigate utilization levels of colorectal, breast and prostate cancer screening in Switzerland, 2) identify potential influencing demographic, insurance, clinical and regional factors, and 3) explore whether the relevant clinical guidelines may additionally contribute to explaining the levels and variation of utilization observed across the screening services. We expected to see less unexplained geographic variation when a service is strongly and routinely recommended (i.e., colonoscopy for colorectal cancer screening), and more variation when screening is not routinely recommended (i.e., prostate cancer screening).

## Methods

### Data source

We analyzed administrative claims data of a major Swiss insurer (Helsana Insurance Group), covering the claims to the mandatory health insurance of approximately 1.2 million people (15% of the Swiss population). The database included information on enrollees’ sociodemographics, such as sex, age and the language region (German, French or Italian), choice of insurance characteristics, and details on reimbursed medical services, provided between January 1, 2014, and December 31, 2014. We excluded enrollees with incomplete insurance coverage or not surviving until the end of 2014, asylum seekers, persons living outside Switzerland, Helsana employees, and persons living in nursing homes and receiving lump-sum reimbursement (which could mask individual medication claims).

Basic health insurance is mandatory in Switzerland and is provided by private insurers. The benefit package is defined nationally, and includes all appropriate and cost-effective inpatient and outpatient services. Persons can choose between different annual deductibles (ranging from 300 to 2,500 Swiss Francs), with higher deductible resulting in lower monthly premiums. Persons can opt for supplementary health insurance (covering some additional healthcare services, such as alternative medicine or dental care) and supplementary inpatient hospital care insurance (adding benefits such as single room accommodation). Persons can also choose a managed care plan for smaller premiums, which requires that a general practitioner or telemedicine provider is the first contact for each new health problem.

### Population

The eligible population was defined separately for each cancer screening service, considering the relevant Swiss and international clinical guidelines. National and European guidelines valid in 2014 were identified through relevant medical societies. National and cantonal reimbursement policies were characterised based on publicly available information (Fig 1).

Colorectal cancer screening was recommended and reimbursed for 50–69 years old persons: either colonoscopy every ten years or fecal occult blood test (FOBT) every two years [12].

Breast cancer screening was recommended and reimbursed in cantons with an organized cantonal screening program: mammography every two years [12]. Women between ages 50–69 years (in cantons of Grisons, St. Gallen) or 50–74 years (in cantons of Bern, Fribourg, Geneva, Jura, Neuchâtel, Thurgau, Vaud, Valais) were systematically invited to participate in 2014 [12]. We analyzed the female population aged 50–74 in this study.

Prostate cancer screening with prostate specific antibody (PSA) test was not recommended routinely, and could be indicated only after considering personal risk factors and preferences in a shared decision making process [9]. Recommended age limits and frequency of screening were not strictly defined, but age from 50 to 70 was mentioned as a reasonable range [9].

We did not exclude persons based on comorbidities or history of cancer. Due to limited clinical data available in the claims datasets, it would not be possible to identify all such persons, and imprecise exclusion criteria could introduce bias. However, we did adjust in the multilevel models for available clinical characteristics.

### Outcome and explanatory variables

In our study, *screening service* implied that a test, suitable for screening, was used for an eligible person, regardless of the indication. This means that tests done for diagnostic or follow-up reasons were also included. This decision was made partly because indications (i.e., screening, diagnostic testing due to certain symptoms present, or follow-up after clinical disease) are not registered in the claims data, and partly because a diagnostic or follow-up test may additionally function as an opportunistic screening.

We classified patients as receiving screening if a related claim was registered in 2014. The screening services analyzed were mammography for breast cancer, PSA testing for prostate cancer, and colonoscopy for colorectal cancer. For colorectal cancer, FOBT is a valid alternative. However, it is complicated to pool both tests together for combined analysis because screening frequency is different (Fig 1) and colonoscopy is also recommended as a follow up for a positive FOBT result. Thus, we restricted our main analysis to colonoscopy, which is used by more persons in Switzerland [19]. FOBT utilization was analyzed separately, and we present the results and their discussion in S2 Appendix.

We considered socio-demographic and clinical characteristics, and area of living at the beginning of 2014, as explanatory variables. Health insurance characteristics analyzed were whether any supplementary health insurance or supplementary hospital insurance was present, whether enrollees had registered with a managed care model, and the chosen level of deductible. Pharmaceutical cost group (PCG) categories derived from outpatient drugs prescribed in 2013 were used as proxies for comorbidities [20]. PCGs representing cancer and inflammatory bowel disease (IBD) were considered as separate binary variables, and the total number of the other PCGs as ordinal variables. We further defined indicators of major colon, breast and prostate diseases, based on inpatient diagnoses and procedures, and outpatient procedures and medications in 2013. Regional variables used were the purchasing power index measured on a zip code level (centered at 100 as the mean value for the Swiss population) and urban vs. rural residence. Cantons were grouped for the analysis of breast cancer screening by the provision of a screening program in 2014. In a sensitivity analysis, cantons were also grouped in this way for analysis of prostate cancer screening variation. Patients were assigned to 106 Swiss MobSpat (“mobilité spatiale”) regions, defined by the Swiss Federal Statistical Office [21], according to their place of residence. MobSpat regions have already been used for research of the geographic variation of healthcare services in Switzerland previously [22].

The specific definitions of clinical variables used are provided in S1 Table.

### Statistical analysis

We first calculated descriptive statistics of the populations grouped by utilization of the applicable screening service. We estimated the overall screening utilization for Switzerland by weighting the cantonal utilization by the total cantonal population in 2014, as the market share of Helsana differs slightly across cantons.

Second, we ran a multilevel multivariable logistic regression model for each screening service, with pre-specified variables as fixed person level effects, and random intercepts for the 106 Swiss MobSpat regions. We included age as a cubic term in the models. The reporting of the marginal effects in multilevel model would have been limited, thus we present the effects from multivariable logistic regression models with the same covariates.

Third, we explored the geographic variation of screening utilization. We first mapped the unadjusted utilization by MobSpat region. Spatial autocorrelation of regional utilization levels was then analyzed with global Moran’s I statistic [23,24]. Moran’s I typically ranges from 1 (neighboring regions are more similar than distant regions) to 0 (spatial distribution of utilization values is random) to -1 (neighboring regions are less similar than distant regions). We then calculated median odds ratios (MOR) for the MobSpat level to estimate the degree of geographic random variation [25,26]. MOR converts the level of variation remaining after multivariable adjustment to the odds ratio (OR) scale, and thus can be directly compared to the fixed-effects. MOR can be interpreted as the median odds of receiving the screening service if an otherwise equal patient would be residing in a different MobSpat region with a higher rate of screening utilization. MOR is always equal or above one because regions of higher utilization are compared to regions of lower utilization. To examine whether clustering was still present in the residuals of the multilevel models, they were aggregated per MobSpat region, mapped, and spatial autocorrelation was again evaluated with the global Moran’s I statistic.

The degree of unexplained geographic variation (MOR and Moran’s I) after the multivariable adjustment was compared between cancer screening services in terms of the associated evidence and guideline recommendations strength (Fig 1).

Statistical analyses were performed with R 3.4.4 [27], STATA 14.2, and MLwiN 3.01 [28] integrated in STATA. Mapping was performed with QGIS 2.18.9 [29], and spatial clustering analysis with GeoDa 1.12 [30]. P values <0.05 were considered statistically significant.

All procedures performed in the study were in accordance with the ethical standards of the 1964 Helsinki declaration and its later amendments. Study data were anonymized before analysis. According to the national ethical and legal regulations, ethical approval was not required for this type of retrospective study. This was confirmed by a waiver of the competent ethics committee (Kantonale Ethikkommission Zürich, dated January 11, 2017).

## Results

### Utilization levels of cancer screening

In the year 2014, Helsana enrollees eligible for the analyses of colorectal cancer, breast cancer and prostate cancer screening comprised 276 387, 178 145 and 145 874 individuals, respectively. Of these, 5.9% received colonoscopy, 20.9% mammography, and 28.4% PSA testing. After weighting by the total cantonal populations, the estimated annual screening probabilities in Switzerland were 8.1% for colonoscopy, 22.3% for mammography and 31.3% for PSA testing. Considering recommended screening intervals (colonoscopy once in ten, mammography once in two years) and based on simple probability-rate-probability conversions (assuming constant screening rates) [31], approximately 57% of the eligible populations would receive colonoscopy, and approximately 40% breast cancer screening, in total.

Socio-demographic, insurance preference and clinical characteristics of the populations are shown in Table 1. The proportions of persons with urban residence, supplementary insurance, managed care, and a high deductible level were similar among persons receiving each cancer screening services, as was the number of PCGs present. In contrast, the screened populations were rather different in terms of linguistic regions. For example, there were more persons from French-speaking regions among persons receiving mammography than among those receiving colonoscopy or PSA testing.

**Table 1:** Characteristics of eligible population receiving cancer screening services in 2014.

	Colorectal (N = 276 387)^a^		Breast (N = 178 145)		Prostate (N = 145 874)	
Screening service provided	No	Yes	No	Yes	No	Yes
N	260010	16377	140882	37263	104516	41358
% of all eligible	94.1	5.9	79.1	20.9	71.6	28.4
Female (%)	134212 (51.6)	8463 (51.7)				
Age (mean (SD))	58.53 (5.84)	59.45 (5.80)	61.22 (7.27)	60.35 (6.97)	58.30 (6.13)	61.25 (6.03)
Purchasing power index on zip code level (mean (SD))	101.62 (22.02)	103.50 (23.48)	101.91 (22.23)	101.14 (22.11)	101.52 (21.87)	102.09 (23.05)
Urban (%)	198011 (76.2)	12964 (79.2)	109070 (77.4)	28976 (77.8)	78072 (74.7)	32110 (77.6)
Language (%)						
German	201483 (77.5)	12704 (77.6)	112107 (79.6)	24453 (65.6)	83972 (80.3)	29951 (72.4)
French	39708 (15.3)	2383 (14.6)	18328 (13.0)	9261 (24.9)	14099 (13.5)	7275 (17.6)
Italian	18819 (7.2)	1290 (7.9)	10447 (7.4)	3549 (9.5)	6445 (6.2)	4132 (10.0)
Supplementary insurance (%)	192895 (74.2)	12568 (76.7)	108376 (76.9)	29191 (78.3)	74447 (71.2)	31712 (76.7)
High deductible (≥500 CHF) (%)	73544 (28.3)	3267 (19.9)	31493 (22.4)	6216 (16.7)	37720 (36.1)	8366 (20.2)
Managed care (%)	132358 (50.9)	8317 (50.8)	70055 (49.7)	19088 (51.2)	53231 (50.9)	20918 (50.6)
Supplementary hosp. ins. (%)	57081 (22.0)	4584 (28.0)	35974 (25.5)	10694 (28.7)	19071 (18.2)	10354 (25.0)
Comorbidities (%)						
0	121697 (46.8)	5771 (35.2)	57733 (41.0)	12691 (34.1)	55868 (53.5)	12586 (30.4)
1	52328 (20.1)	3674 (22.4)	29616 (21.0)	8528 (22.9)	18727 (17.9)	8942 (21.6)
2	38632 (14.9)	2885 (17.6)	22033 (15.6)	6546 (17.6)	14110 (13.5)	8840 (21.4)
3+	47353 (18.2)	4047 (24.7)	31500 (22.4)	9498 (25.5)	15811 (15.1)	10990 (26.6)
PCG Cancer	2854 (1.1)	334 (2.0)	1891 (1.3)	903 (2.4)	865 (0.8)	537 (1.3)
PCG IBD	1253 (0.5)	290 (1.8)				
Major colon disease (%)	804 (0.3)	254 (1.6)				
Major breast disease (%)			1192 (0.8)	2185 (5.9)		
Major prostate disease (%)					658 (0.6)	1585 (3.8)
In canton with program^b^			50739 (36.0)	19193 (51.5)		

SD–standard deviation, CHF–Swiss francs, Supplementary hosp.ins.–supplementary inpatient hospital care insurance, PCG–pharmaceutical cost group, IBD–inflammatory bowel disease.

^a^ Eligible persons who received FOBT (fecal occult blood test) but not colonoscopy are excluded. Only colonoscopy is considered as screening service here. FOBT is reviewed separately in S2 Appendix.

^b^ Cantonal-level coordinated breast cancer screening program.

### Multilevel modelling of cancer screening utilization

Multilevel logistic regression results for the utilization of each cancer screening service are shown in Table 2. Persons living in areas with higher purchasing power and urban areas were more likely to receive screening. French- and Italian-speaking regions were more likely than German regions to receive breast and prostate, but not colorectal cancer screening. Higher deductible level was consistently associated with smaller odds of screening (CHF 2500 vs CHF 300: 0.63 [0.60–0.67] for colorectal cancer screening, 0.68 [0.65–0.71] for breast cancer screening, 0.56 [0.54–0.59] for prostate cancer screening utilization), while supplementary health insurance, supplementary inpatient hospital care insurance and participation in a managed care plan were associated with more screening.

**Table 2:** Multilevel model estimates (odds ratio) and spatial clustering analysis for cancer screening services utilization in 2014.

	Colorectal	Breast	Prostate
Female	0.93 [0.90–0.96]	N/A	N/A
Age	0.17 [0.17–0.17]	0.15 [0.15–0.15]	1.17 [1.17–1.18]
Age^2^ (age squared)	1.03 [1.03–1.03]	1.03 [1.03–1.03]	1.00 [1.00–1.00]
Age^3^ (age cubed)	1.00 [1.00–1.00]	1.00 [1.00–1.00]	1.00 [1.00–1.00]
Purchasing power index	1.25 [1.16–1.38]	1.09 [1.01–1.18]	1.20 [1.12–1.28]
Urban	1.07 [1.02–1.12]	1.06 [1.03–1.11]	1.10 [1.07–1.14]
Language			
German	Reference	Reference	Reference
French	0.86 [0.79–0.94]	1.65 [1.50–1.81]	1.38 [1.25–1.53]
Italian	1.11 [0.98–1.27]	1.69 [1.43–2.01]	1.50 [1.28–1.74]
Supplementary insurance	1.05 [1.00–1.10]	1.14 [1.10–1.17]	1.18 [1.14–1.22]
Deductible level, CHF			
300	Reference	Reference	Reference
500	0.92 [0.88–0.95]	0.93 [0.90–0.96]	0.91 [0.89–0.94]
1000	0.81 [0.75–0.88]	0.82 [0.77–0.88]	0.74 [0.70–0.77]
1500	0.73 [0.68–0.78]	0.74 [0.71–0.78]	0.62 [0.60–0.65]
2000	0.63 [0.54–0.72]	0.68 [0.60–0.77]	0.60 [0.55–0.66]
2500	0.63 [0.60–0.67]	0.68 [0.65–0.71]	0.56 [0.54–0.59]
Managed care	1.12 [1.08–1.15]	1.13 [1.10–1.16]	1.13 [1.11–1.15]
Supplementary hospital care insurance	1.34 [1.29–1.40]	1.29 [1.25–1.32]	1.36 [1.33–1.40]
Comorbidities			
0	Reference	Reference	Reference
1	1.30 [1.24–1.36]	1.26 [1.22–1.30]	1.66 [1.61–1.71]
2	1.31 [1.25–1.38]	1.30 [1.25–1.35]	1.90 [1.84–1.95]
3+	1.45 [1.38–1.52]	1.30 [1.25–1.34]	1.83 [1.79–1.89]
PCG Cancer	1.37 [1.21–1.54]	1.14 [1.03–1.24]	0.96 [0.89–1.04]
PCG IBD	2.81 [2.47–3.19]	N/A	N/A
Major colon disease	3.51 [3.03–4.06]	N/A	N/A
Major breast disease	N/A	7.44 [6.90–8.00]	N/A
Major prostate disease	N/A	N/A	3.75 [3.54–3.99]
In canton with program^a^	N/A	1.80 [1.66–1.97]	N/A
**Spatial variation statistics**			
MOR	1.16 [1.12–1.20]	1.20 [1.16–1.25]	1.24 [1.20–1.30]
Moran’s I of raw utilization	0.216 (p<0.001)	0.621 (p<0.001)	0.552 (p<0.001)
Moran’s I of residuals	0.083 (p = 0.074)	0.070 (p = 0.104)	0.492 (p<0.001)

CHF–Swiss francs, PCG–pharmaceutical cost group, IBD–inflammatory bowel disease, MOR–median odds ratio.

The comorbidities variable did not include PCG cancer and PCG IBD. Odds ratio estimates in grey are not statistically significantly different from 1. For colorectal cancer screening modelling, only colonoscopy is considered as the outcome of interest. FOBT (fecal occult blood test) and the combination of both tests is excluded and is reviewed separately in S2 Appendix.

^a^ Cantonal-level coordinated breast cancer screening program.

The presence of PCG comorbidities and specific diseases was associated with a higher probability of receiving screening. Age had a non-linear effect on screening probability (marginal effects depicted in S1 Appendix). Residing in a canton with a coordinated program was associated with an OR for breast cancer screening of 1.80 [1.66–1.97]. In the model of prostate cancer screening utilization, the presence of a breast cancer screening program in the canton of residence resulted in a significant OR of 1.15 [1.07–1.25], with other effect estimates remaining stable.

### Geographic pattern of screening utilization

**Fig 2:**
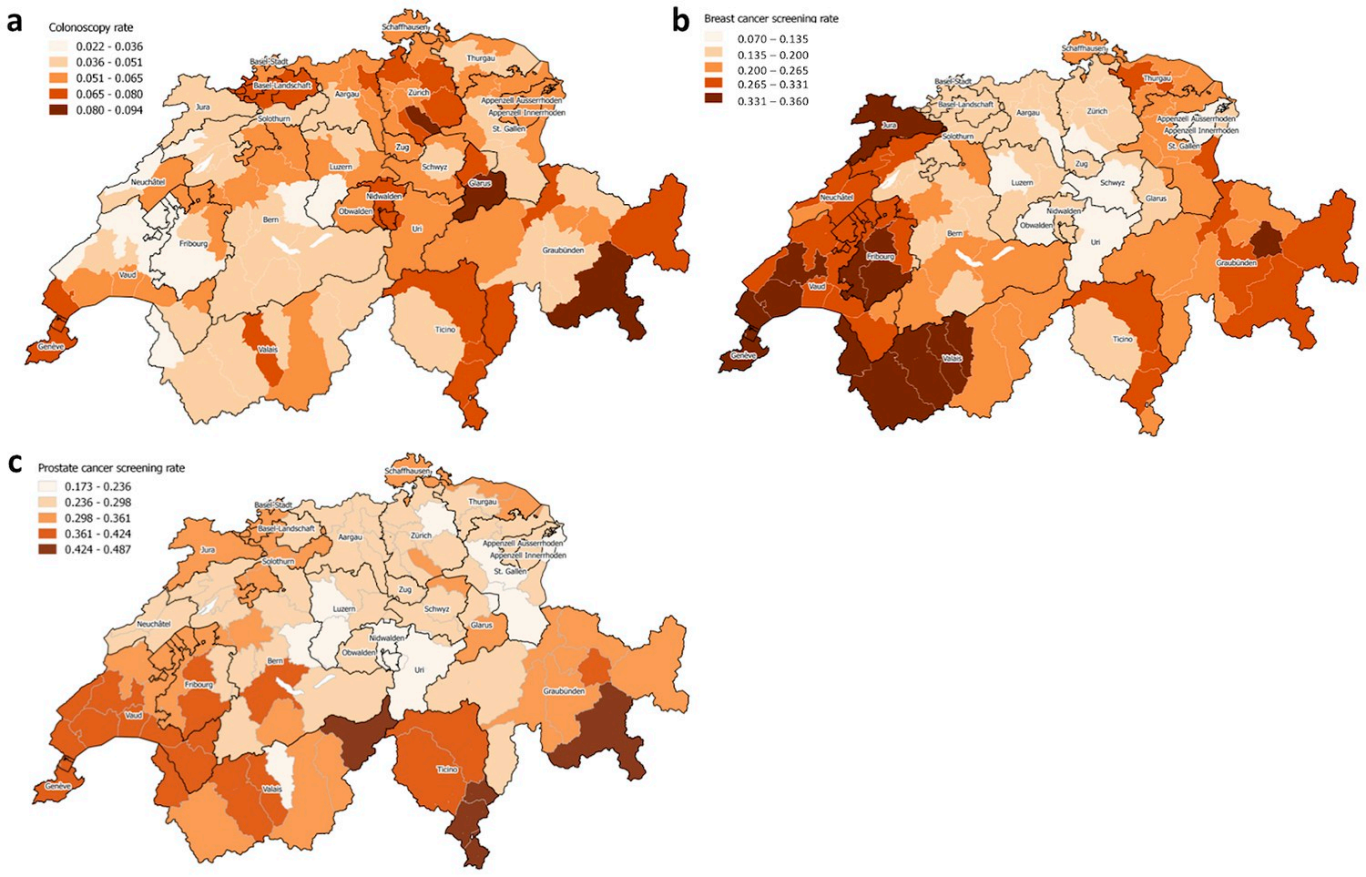
Raw utilization of cancer screening services in eligible population in Switzerland in 2014. A–colorectal cancer screening, B–breast cancer screening, C–prostate cancer screening.

Spatial clustering was observed in the raw regional utilization of all screening services (Fig 2), with stronger clustering for breast (Moran’s I 0.62, p<0.001) and prostate (0.55, p<0.001) than for colorectal cancer screening (0.22, p<0.001) (Table 2). Western and eastern regions of Switzerland had higher raw mammography utilization, while PSA test utilization was somewhat higher in the west and south.

**Fig 3:**
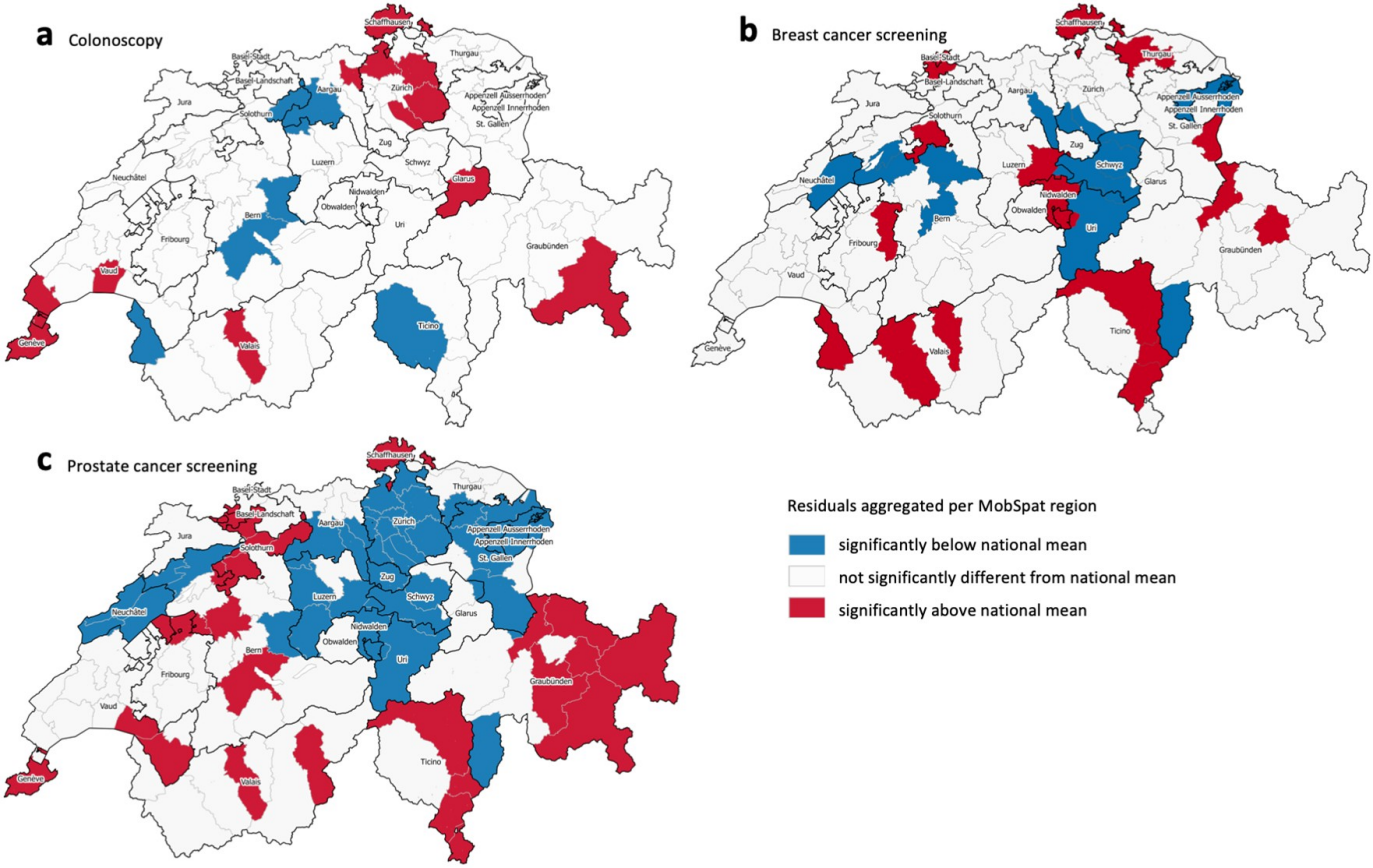
Multilevel models’ regional residuals of cancer screening services utilization, significantly different from national mean. A–colorectal cancer screening, B–breast cancer screening, C–prostate cancer screening.

The MOR after multilevel modelling for the regional level was 1.24 [1.20–1.30] for prostate cancer screening and slightly smaller for breast (1.20 [1.16–1.25]) and colorectal (1.16 [1.12–1.20]) cancer screening. Spatial clustering of multilevel model regional level residuals of screening utilization (Fig 3) was insignificant for colorectal and breast cancer screening, but high for prostate cancer screening (Moran’s I 0.49, p<0.001). Regions with residuals of prostate cancer screening utilization indicating significantly higher use were situated mostly in the west and south-east of Switzerland (Fig 2). Adding the presence of breast cancer screening program in the model of prostate cancer screening utilization slightly reduced the spatial clustering (Moran’s I) of the model’s regional residuals.

## Discussion

The absolute estimated levels of colorectal, breast and prostate cancer screening annual utilization in Switzerland in 2014 were 8.1% (colonoscopy), 22.3% (mammography) and 31.3% (PSA testing), respectively. Insurance characteristics, such as lower deductibles, supplementary insurance, being enrolled in a managed care plan and presence of comorbidities were associated with higher screening utilization. Cantons with organized breast cancer screening programs were associated with more breast cancer screening, whereas the effect of language was inconsistent across the services. Geographic variation, as reflected in MOR, was highest for prostate and lowest for colorectal cancer screening. We observed significant geographic clustering in prostate cancer screening even after influencing factors had been controlled for, signaling potentially unwarranted variation.

We estimated that approximately 57% of the eligible population would receive colorectal cancer screening and 40% breast cancer screening, within ten and two years respectively, as recommended by guidelines (such an estimate was not possible for prostate cancer screening, as the recommended testing frequency is personalized). Compared to the rates of screening in European countries with organized programs, our estimate for colorectal screening was higher than the reported average of approximately 20% [32], and the estimate for breast cancer screening lower than the reported average of approximately 50% [33].

We estimated higher screening utilization in Switzerland than other studies. In a study of primary care patients within the Swiss Sentinel Surveillance Network in 2017, 41% of patients aged 50 to 75 reported colonoscopy in the last ten years [19]. Only 13.3% of women aged 50 to 69 reported mammography in the last 12 months in the Swiss Health Survey in 2012 [16]. In a voluntary database of routine electronic medical records of Swiss primary care practices, 11.7% of men aged 55 to 75 without prostate disease or symptoms received PSA testing in 2010–2017 [15]. Screening utilization estimates may differ between these and our study partly due to differences in design, such as the age range of included persons eligible for screening. Also, we did not exclude potentially indicated (diagnostic and follow-up) tests. Information collection methods were also different. Whereas we used claims data, the other studies used self-reported information or electronic medical records. Potentially, self-reported data could be prone to information, recall and social desirability biases, and electronic health records at the primary care practice could miss tests done elsewhere, e.g. by a urologist. Finally, the year of data collection differed across the previous studies from 2010 to 2017, and the observation time window from one to ten years.

### Effect of explanatory variables

The effect of explanatory variables on screening utilization reflects different barriers and facilitators of access to care. Persons with comorbidities, as reflected by the PCGs and clinical indicators of disease, were more likely to be screened. PCGs and disease indicators signal not only worse health, but also more contact with healthcare services providers. In fact, healthcare services utilization (such as a recent visit to a physician) has been associated with higher screening rates [14]. In contrast to this, worse self-reported health or comorbidities were associated with lower odds of screening [14], even though the effect of different comorbidities might not be the same [34]. Thus, being assigned to PCG or other indicators of diseases from the claims data could constitute both barriers (poor health) and facilitators (receiving healthcare services) to screening. Additionally, more diagnostic and follow-up tests are provided for less healthy people, which would have been observed as screening services in our study. Finally, persons with poorer health status often choose lower deductibles, but also use more health services–leading to a mix of barriers and facilitators.

Lower deductibles, supplementary insurance and urban or wealthier residence were consistently associated with more screening. Health insurance with higher deductibles has been reported to lead to foregoing care in Switzerland, and thus constitute a financial barrier [35]. Participation in a managed care model (for more details see [36]), was associated with more screening–potentially as the care provided is more coordinated and guideline-compliant, or as this model is selected by more health aware persons. Urban residence, better access to healthcare (e.g., shorter distance and higher physician density) [13], as well as higher socioeconomic status or income have been shown to increase the odds of screening both at individual and regional levels [37–39]. Self-reported barriers to screening utilization include lack of awareness, negative attitudes towards screening and socioeconomic factors, and some of the facilitators are public education and physician recommendation [40]. These barriers are indeed partly reflected in our observed association of screening utilization with sociodemographic and insurance characteristics.

Cantonal breast cancer screening programs were a strong driver of screening utilization (Table 2), an effect also observed by Fenner et al. [16]. The effect of language region on screening utilization might reflect differences in acceptance or local health promotion programs. However, the effect of specific language regions was not consistent across screening services (Table 2). In previous studies, Braun et al. found no effect of language region on the utilization of colorectal cancer screening [19], whereas Eichholzer et al. observed a higher utilization of breast cancer screening in French speaking cantons of Switzerland [18].

### Geographic variation within and across screening services

We selected three cancer screening services reflecting different degrees of certainty in terms of their evidence of benefit-to-harm balance, impact on health outcomes, and clinical guidelines recommending them. We expected to observe a correlation with different degrees of regional variation (MOR) and, potentially, different degrees of unexplained spatial clustering patterns (Moran’s I). The highest MOR of 1.24 was observed for prostate cancer screening, which is not routinely recommended. The MobSpat-level residuals in the multilevel prostate cancer model were the only ones that remained significantly spatially autocorrelated, signaling that a relevant part of regional variation was not explained (Fig 3). Breast cancer screening, which is recommended but debated, had slightly lower residual geographic variation, and the variation was lowest for colorectal cancer screening, which is consistently recommended in guidelines. The interpretation of this trend needs to consider that screening services also differ by other characteristics, not considered in our models. For example, they are delivered by different specialists (e.g., PSA test often by primary care physicians and colonoscopy by gastroenterologists); reimbursement policies are different (e.g., screening colonoscopy covered by mandatory health insurance while screening mammography only covered, and exempt from deductible, in cantons with a screening program); the eligible populations are of different sexes. Still, this is the first study indicating a relationship between the strength of recommendations and geographic variation of screening utilization, warranting further exploration.

The unexplained geographic variation seen in prostate cancer screening could have several reasons. First, prostate cancer screening recommendation in both Swiss [9] and European guidelines [10] are conditional, and could be considered *weak* and sensitive to patient preferences, according to the GRADE approach [41]. This recommendation could be interpreted as *discouraging* routine use, but it could also be interpreted as *encouraging* screening after a shared-decision making process. Second, even after adjusting for language and other covariates, the observed clusters of high utilization partly coincided with the presence of breast cancer screening programs (Fig 2 and S1 Fig). In fact, adding an indicator for the presence of a cantonal breast cancer screening program to the model resulted in a significant OR and slightly reduced spatial clustering (Moran’s I) of the model’s regional residuals. This could mean that breast cancer screening programs encourage gender-specific or any cancer screening more broadly. It has already been observed that regional utilization of prostate cancer screening could be partly explained by the utilization of another cancer screening [42]. However, we did not observe a comparable pattern for colorectal cancer screening. Third, we may have missed important locally specific influencing factors, such as patient and provider preferences. In addition, local spill-over effects may be taking place among neighboring MobSpat regions, that is, patient and provider preferences may be more similar or even stimulate each other across neighboring MobSpat regions. However, we believe that the effect estimates of the currently included covariates would not change significantly, as they were consistent with the models of colorectal and breast cancer screening utilization.

### Limitations

Our study has several limitations. First, we used claims data from a single, albeit largest in Switzerland, health insurer. Although the population of Helsana insured persons is slightly older than the general Swiss population [43], the benefits package of the mandatory health insurance is defined at the federal level, and patients can choose their healthcare providers regardless of insurance provider. Second, claims data have limited clinical information, such as outpatient diagnoses, and lack past claims if the patient was previously insured by another company. For this reason, we could not distinguish diagnostic and follow-up use of screening services. Clinically indicated and screening tests could be associated with different barriers and facilitators. In our study, a clinical indicator of related diseases was associated with more screening. This association could signal that patients with such diseases receive more diagnostic and follow-up tests, not screening. Thus, not excluding clinically indicated tests could result in an overestimation of screening utilization. On the other hand, our primary interest was the variation in utilization within and between the screening services. We would not expect a sufficiently large degree of geographic variation in, e.g., the presence of prostate disease and symptoms, to explain the observed clustering of PSA test utilization. Third, services paid out-of-pocket were not recorded. This could underestimate the utilization of less expensive screening procedures (PSA and FOBT testing), whereas mammography and colonoscopy are not likely to be paid out of pocket. Finally, we could not analyze the influence of screening service provider, which could be a significant driver of regional variation [44].

### Further research implications

This study raises further questions. As new cantons are introducing screening programs, patients may become more aware of reimbursement policies and guidelines may change, we plan to investigate the effect on screening utilization with multiple temporal cross-sections of the following years. For example, the mean level of prostate cancer screening utilization was shown to have changed in Switzerland following the publication of trial results and guidelines [15], but the change in geographic variation is not known. The regional density of relevant specialty physicians could explain some further variation. Diagnostic and screening use of the services could be better differentiated and explained by specific facilitators and barriers, by linking clinical information or running subgroup analyses. In general, merging claims data with clinical or patient preferences data would likely increase the explanatory power of the models. As some regional variation remains unexplained, we can only hypothesize if it is related to further regional, provider or patient characteristics.

## Conclusions

We observed that the absolute levels of colorectal, breast and prostate cancer screening annual utilization in 2014 in Switzerland were 8.1%, 22.3% and 31.3%, respectively. Insurance characteristics, such as low deductibles, supplementary health insurance and managed care plan, were consistently associated with higher screening utilization levels, potentially signaling modifiable barriers and facilitators. Breast cancer screening programs were shown to be successful in promoting screening utilization–possibly because they increase awareness of screening benefits and reduce financial barriers. After controlling for explanatory variables, spatial clustering, indicating potentially unwarranted variation, was present only in prostate cancer screening, which is not recommended routinely without explicit shared decision-making. Prostate cancer screening utilization also had the highest regional level variation, while colorectal cancer screening had the lowest, hinting at the potential role of strong recommendations and evidence in decreasing the variation of services utilization. The reasons for the variation of the prostate cancer screening utilization could be further investigated by adding provider level characteristics and patient preference information. This first cross-comparison of different cancer screening patterns indicates that the strength of guidelines, mediated by the implementation of specific health policies facilitating screening, may indeed influence the degree of variation.

## Supporting information

S1 Table Definitions of clinical variables used in multilevel models. (PDF)

S1 Appendix Predicted marginal effect of age on cancer screening (age^3^) in the logistic regression models. (PDF)

S2 Appendix Fecal occult blood test (FOBT) for colorectal cancer screening analysis. (PDF)

S1 Fig Swiss cantons with a breast cancer screening program in 2014. (PDF)
