# Supplementary material for: Variation of colorectal, breast and prostate cancer screening activity in Switzerland: Influence of insurance, policy and guidelines
Source: PLoS One. 2020 Apr 16;15(4):e0231409. doi: 10.1371/journal.pone.0231409 (PMC7162274; doi:10.1371/journal.pone.0231409)
Supplement: S1 Table — (PDF) [file pone.0231409.s001.pdf]

Agne Ulyte, Wenjia Wei, Holger Dressel, Oliver Gruebner, Viktor von Wyl, Caroline Bähler, Eva Blozik, Beat Brüngger, Matthias Schwenkglenks. Variation of colorectal, breast and prostate cancer screening activity in Switzerland: influence of insurance, policy and guidelines

S1 Table Definitions of clinical variables used in multilevel models

**Name of variable**	**Definition: any of the codes present**
**Screening services (in 2014)**	
Colonoscopy	DRG: G48%<br>CHOP: 45.23, 45.25, 48.29.1%, 48.29.2%<br>TM Kapitel: 19.06
Fecal occult blood test (FOBT)	Ana: 1583.00, 1583.01
Mammography	TM: 39.1310, 39.1320, 39.1307, 39.1308, 39.1300, 39.1305, 39.1306<br>TZ
Prostate specific antigen (PSA) test	Ana: 1626.00
**Specific comorbidities (in 2013)**	
Major colon disease	ICD: C18%, C19%, C20%, C21%, Z80.0, Z85.0, D01%, Z93.3, K63.5, Z93.2, D12%, K52%, K51%, K50%<br>CHOP: 46.1%, 45.4%, 45.7%, 45.8%<br>TM Kapitel: 20.08
Major breast disease	ICD: C50%, Z80.3, D05%, D24%, D48.6<br>DRG: J07Z, J14%, J16Z, J18Z, J23Z, J25Z, J26Z, J62%<br>CHOP: 85%<br>TM Kapitel: 23.02, 39.02.05<br>TM: 23.0110, 23.0120<br>ATC: L02BG03, L02BG06, L02BA03, L02AE03, L02BG04, L01XE33, L01XC13, L01XE42, L02BA01, L02BA02, L01XC03, L01XC14
Major prostate disease	ICD: C61, D07.5, N41%, D29.1, N40<br>CHOP: 60.6%, 60.9%, 60.11, 60.12, 60.5%, 60.4, 60.3, 60.2%<br>TM: 21.2120, 21.2170, 21.2180, 21.2190, 21.2125<br>ATC: L02AE03, L02AE05, L02AE02, L02AE04, L02BB%, L02BG%, L02BX%

Note. A single code from the ones listed in the table is sufficient to identify screening service or the comorbidity.
Abbreviations:
Ana – Analysenliste, Swiss outpatient laboratory test codes; ATC - Anatomical Therapeutic Chemical Classification System, code and quantity of a prescription drug; CHOP - Schweizerische Operationsklassifikation, a classification of inpatient procedures; DRG - Swiss Diagnosis Related Groups, a classification of inpatient cases, based on diagnoses, procedures and other clinical information; ICD - International Classification of Diseases, 10th revision, German Modification, codes for primary and secondary diagnoses for each hospitalization episode of an inpatient; TM – Tarmed, Swiss classification of outpatient procedures and services; TM Kapitel – Tarmed chapter codes; TZ – Tarifziffer, further codes representing reimbursement of screening services within cantonal breast cancer screening programs.
