# Supplementary material for: Variation of colorectal, breast and prostate cancer screening activity in Switzerland: Influence of insurance, policy and guidelines
Source: PLoS One. 2020 Apr 16;15(4):e0231409. doi: 10.1371/journal.pone.0231409 (PMC7162274; doi:10.1371/journal.pone.0231409)
Supplement: S1 Appendix — (PDF) [file pone.0231409.s002.pdf]

S2 Appendix Predicted marginal effect of age on cancer screening (age^3) in the logistic regression models
A – colorectal cancer screening, B – breast cancer screening, C – prostate cancer screening.

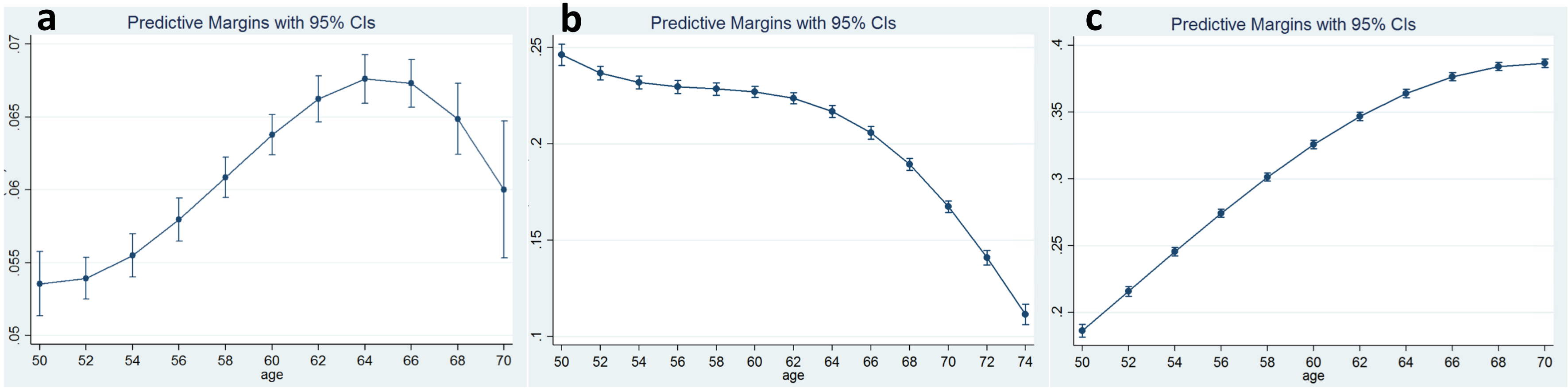

Note. The estimation of marginal effects in the multilevel model is limited. Therefore, marginal effects of age in the logistic regression models, rather than the multilevel models, are reported here.
The probability of colonoscopy increased with age until approximately 65 years and decreased in older age, the probability of mammography was highest for age 50-65 years and decreased thereafter, and the probability of PSA testing increased steadily across the investigated age range.
