# Supplementary material for: Variation of colorectal, breast and prostate cancer screening activity in Switzerland: Influence of insurance, policy and guidelines
Source: PLoS One. 2020 Apr 16;15(4):e0231409. doi: 10.1371/journal.pone.0231409 (PMC7162274; doi:10.1371/journal.pone.0231409)
Supplement: S2 Appendix — (PDF) [file pone.0231409.s003.pdf]

S3 Appendix Fecal occult blood test (FOBT) for colorectal cancer screening analysis

Methods. In addition to colonoscopy, we ran a supplementary analysis of FOBT as a screening service of colorectal cancer. For this purpose, eligible persons receiving the specified screening service (colonoscopy, FOBT, or both) were considered as screened, and eligible persons not receiving any of the tests as not screened. While the outcome of interest was colonoscopy in the main analysis, it was any of FOBT, FOBT and colonoscopy, or FOBT and/or colonoscopy, in the supplementary analysis. The tests were examined both separately and in combination, as separate or combined use may be seen in different populations of patients. For example, the population with FOBT and colonoscopy would be more likely to include persons screened positive with FOBT, because colonoscopy is required as a follow up procedure.

Results of the multilevel model and geographic pattern analysis. In the main analysis, we considered colorectal cancer screening with colonoscopy. Corresponding analyses of screening with FOBT, as well as of the combinations of FOBT and colonoscopy, or FOBT and/or colonoscopy, are presented here. The estimated effects of the predictor variables in the multilevel models for utilization of FOBT or FOBT and/or colonoscopy were similar to the effects in the model for utilization of colonoscopy, except for the estimated effect of language, categorization into the cancer PCG, categorization into the IBD PCG and indicator of major colon disease. The latter three variables were associated with less FOBT utilization. The probability of FOBT utilization increased with age. Marked geographic variation of FOBT utilization was present (multilevel model MOR for the regional level 1.52, Moran's I 0.17, p=0.003).

The characteristics of patients receiving colonoscopy and/or FOBT, the estimates of the multilevel model effects, and the results of the geographic analysis are provided below.

S3a Table Characteristics of patients receiving colorectal cancer screening as colonoscopy, fecal occult blood test or as both services (N total = 283 422)

	No test	Only Colonoscopy	Only FOBT	Colonoscopy **and** FOBT	Colonoscopy **or** FOBT
N (%)	260010	16377 (5.9)	6095 (2.3)	940 (0.4)	23412 (8.3)
Female	134212 (51.6)	8463 (51.7)	3015 (49.5)	463 (49.3)	11941 (51.0)
Age (mean (SD))	58.53 (5.84)	59.45 (5.80)	60.15 (5.74)	60.30 (5.58)	59.67 (5.78)
Purchasing power index on zip code level (mean (SD))	101.62 (22.02)	103.50 (23.48)	100.16 (21.13)	100.00 (20.56)	102.49 (22.83)
Urban (%)	198011 (76.2)	12964 (79.2)	4764 (78.2)	716 (76.2)	18444 (78.8)
Language (%)					
German	201483 (77.5)	12704 (77.6)	4630 (76.0)	742 (78.9)	18076 (77.2)
French	39708 (15.3)	2383 (14.6)	724 (11.9)	82 (8.7)	3189 (13.6)
Italian	18819 (7.2)	1290 (7.9)	741 (12.2)	116 (12.3)	2147 (9.2)
Supplementary insurance (%)	192895 (74.2)	12568 (76.7)	4802 (78.8)	732 (77.9)	18102 (77.3)
High deductible (≥500 CHF) (%)	73544 (28.3)	3267 (19.9)	1109 (18.2)	182 (19.4)	4558 (19.5)
Managed care (%)	132358 (50.9)	8317 (50.8)	3229 (53.0)	479 (51.0)	12025 (51.4)
Supplementary hospital care insurance (%)	57081 (22.0)	4584 (28.0)	1486 (24.4)	242 (25.7)	6312 (27.0)
Comorbidities (%)					
0	121697 (46.8)	5771 (35.2)	2016 (33.1)	327 (34.8)	8114 (34.7)
1	52328 (20.1)	3674 (22.4)	1427 (23.4)	193 (20.5)	5294 (22.6)
2	38632 (14.9)	2885 (17.6)	1213 (19.9)	173 (18.4)	4271 (18.2)
3+	47353 (18.2)	4047 (24.7)	1439 (23.6)	247 (26.3)	5733 (24.5)
PCG Cancer	2854 (1.1)	334 (2.0)	50 (0.8)	15 (1.6)	399 (1.7)
PCG IBD	1253 (0.5)	290 (1.8)	20 (0.3)	4 (0.4)	314 (1.3)
Major colon disease (%)	804 (0.3)	254 (1.6)	12 (0.2)	8 (0.9)	274 (1.2)

FOBT – fecal occult blood test; SD – standard deviation; PCG – pharmaceutical cost group; IBD – inflammatory bowel disease.

S3b Table Multilevel model effect estimates (odds ratios) and spatial clustering analysis for colorectal cancer screening test utilization in 2014

	Colonoscopy **or** FOBT	Colonoscopy	FOBT
Female	0.91 [0.88-0.93]	0.93 [0.90-0.96]	0.86 [0.82-0.90]
Age	0.25 [0.25-0.26]	0.17 [0.17-0.17]	0.66 [0.65-0.66]
Age^2	1.02 [1.02-1.02]	1.03 [1.03-1.03]	1.01 [1.01-1.01]
Age^3	1.00 [1.00-1.00]	1.00 [1.00-1.00]	1.00 [1.00-1.00]
Purchasing power index	1.22 [1.14-1.32]	1.25 [1.16-1.38]	1.31 [1.11-1.57]
Urban	1.07 [1.03-1.11]	1.07 [1.02-1.12]	1.10 [1.01-1.19]
Language			
German	Reference	Reference	Reference
French	0.89 [0.81-0.97]	0.86 [0.79-0.94]	1.00 [0.80-1.25]
Italian	1.25 [1.09-1.43]	1.11 [0.98-1.27]	1.31 [0.95-1.86]
Supplementary insurance	1.09 [1.05-1.12]	1.05 [1.00-1.10]	1.23 [1.14-1.31]
Deductible level, CHF			
300	Reference	Reference	Reference
500	0.92 [0.89-0.95]	0.92 [0.88-0.95]	0.93 [0.87-0.98]
1000	0.79 [0.74-0.85]	0.81 [0.75-0.88]	0.76 [0.66-0.86]
1500	0.72 [0.67-0.76]	0.73 [0.68-0.78]	0.67 [0.60-0.75]
2000	0.63 [0.55-0.71]	0.63 [0.54-0.72]	0.60 [0.47-0.77]
2500	0.62 [0.58-0.65]	0.63 [0.60-0.67]	0.57 [0.52-0.64]
Managed care	1.13 [1.10-1.16]	1.12 [1.08-1.15]	1.17 [1.11-1.23]
Supplementary hospital care insurance	1.26 [1.22-1.30]	1.34 [1.29-1.40]	1.06 [0.99-1.13]
Comorbidities			
0	Reference	Reference	Reference
1	1.33 [1.28-1.38]	1.30 [1.24-1.36]	1.44 [1.34-1.55]
2	1.37 [1.32-1.43]	1.31 [1.25-1.38]	1.54 [1.42-1.66]
3+	1.46 [1.41-1.52]	1.45 [1.38-1.52]	1.49 [1.38-1.60]
PCG Cancer	1.16 [1.04-1.29]	1.37 [1.21-1.54]	0.60 [0.45-0.78]
PCG IBD	2.17 [1.90-2.48]	2.81 [2.47-3.19]	0.59 [0.36-0.89]
Major colon disease	2.78 [2.41-3.19]	3.51 [3.03-4.06]	0.56 [0.30-0.92]

Spatial variation statistics			
MOR	1.15 [1.12-1.19]	1.16 [1.12-1.20]	1.52 [1.42-1.65]
Moran's I of raw rate	0.083 (p=0.072)	0.216 (p<0.001)	0.119 (p=0.025)
Moran's I of residuals	-0.074 (p=0.147)	0.083 (p=0.074)	0.170 (p=0.003)

FOBT – fecal occult blood test, CHF – Swiss francs, PCG – pharmaceutical cost group, IBD – inflammatory bowel disease, MOR – median odds ratio.
Odds ratio estimates in grey are not statistically significantly different from 1.

S3c Figure Marginal effect of age on colorectal cancer screening modality (age^3) in the logistic regression models
A – Colonoscopy and/or FOBT, B – Colonoscopy, C – FOBT.
FOBT – fecal occult blood test.

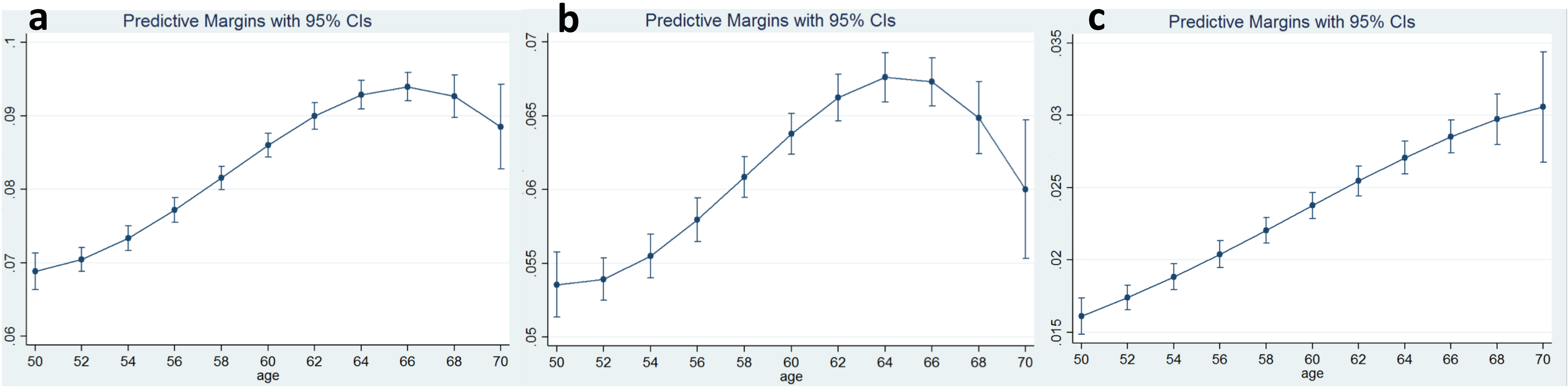

Note. The estimation of marginal effects in the multilevel model is limited. Therefore, marginal effects of age in the logistic regression models, rather than the multilevel models, are reported here.

S3d Figure Raw rate of colorectal cancer screening modality utilization in eligible population in Switzerland
A – Colonoscopy, B – fecal occult blood test (FOBT).

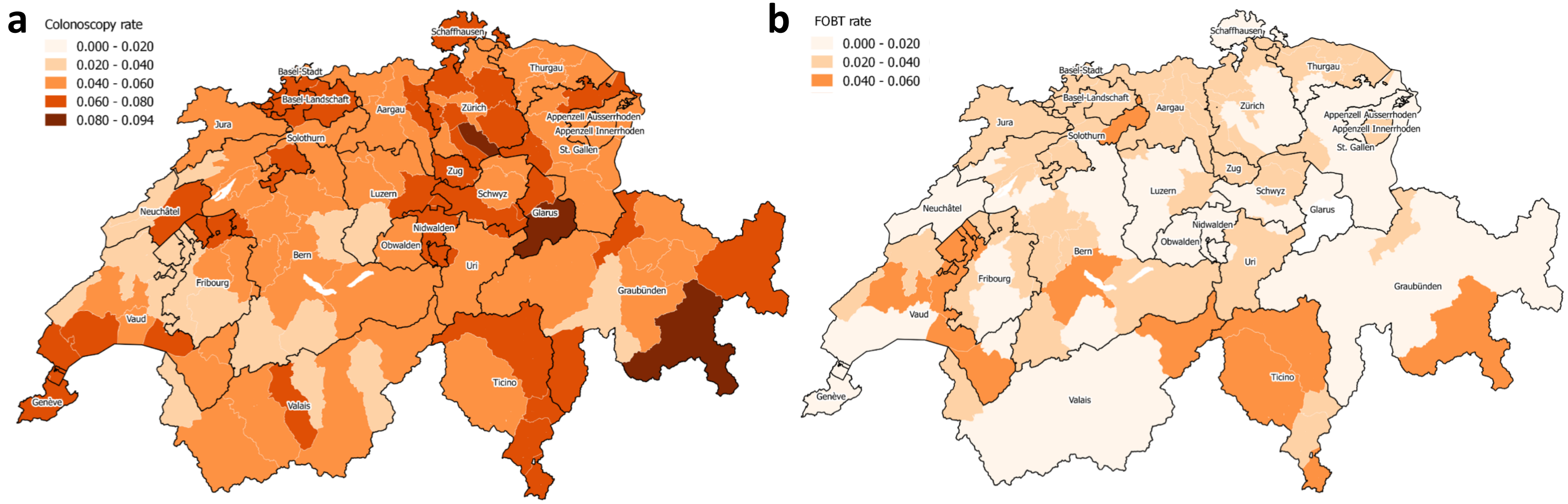

Fig 4a Colorectal cancer screening with colonoscopy or FOBT: multilevel models' regional residuals of cancer screening utilization, significantly different from national mean

A – Colonoscopy, B – fecal occult blood test (FOBT).

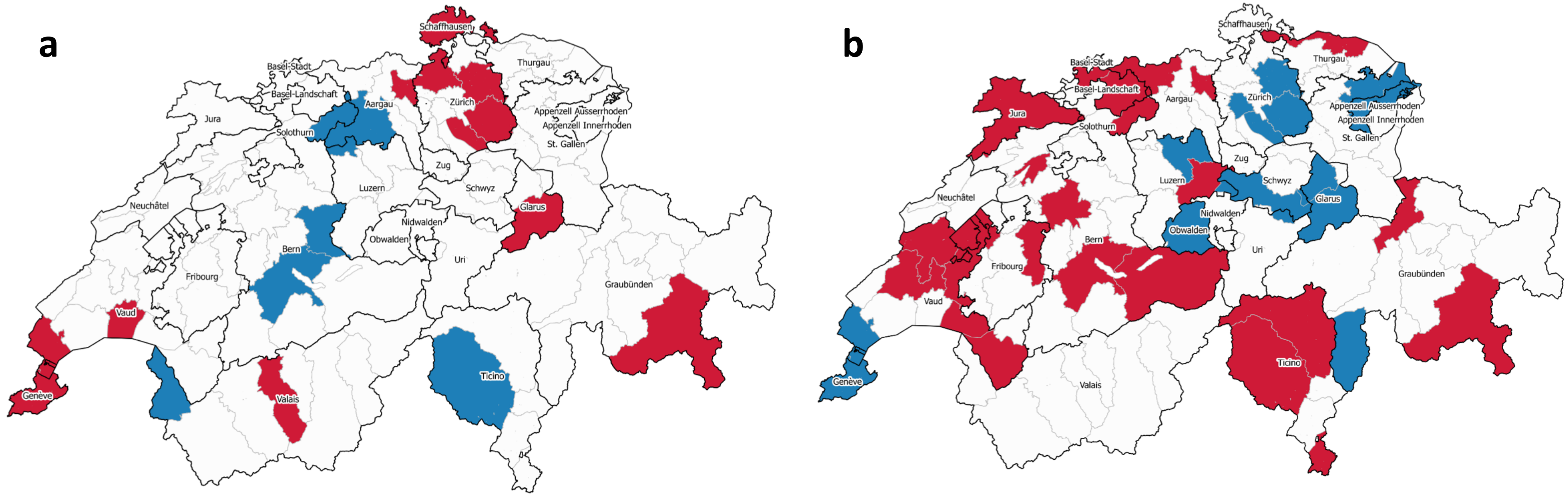

Discussion. In contrast to colonoscopy, FOBT as a colorectal cancer screening modality was associated with high regional variation (MOR of 1.52) (S3b Table). Even though both colonoscopy and FOBT are used for colorectal cancer screening and are associated with excellent outcomes, the mortality benefits, potential harms, sensitivity and specificity profiles are different [1]. Colonoscopy requires a gastroenterologist or other specialist to perform it, and is recommended only every ten years, in contrast to FOBT (every two years). The choice of tests is also associated with difference patient preferences [2]. FOBT and colonoscopy are potentially preferred by different persons, which we regard as reflected in the differential effects of some covariates in the multilevel models. For example, persons classified into the cancer or IBD PCGs and with the indicator of major colon diseases were more likely to use colonoscopy, but less likely to use FOBT. This is also partly explained as colonoscopy is used more for diagnostic and follow-up purposes, whereas FOBT is used mostly for screening. On the other hand, the absolute utilization of FOBT was lower than colonoscopy, especially considering that it should be done every two years. As FOBT is much cheaper and more likely to be paid out-of-pocket, it may be more often missed in the claims data. Thus, the pattern of FOBT utilization as reflected in our study could potentially be more biased.

Bibliography

1. Bibbins-Domingo K, Grossman DC, Curry SJ, Davidson KW, Epling JW, García FAR, et al. Screening for Colorectal Cancer. JAMA. 2016;315(23):2564.
2. Marshall DA, Johnson FR, Phillips KA, Marshall JK, Thabane L, Kulin NA. Measuring Patient Preferences for Colorectal Cancer Screening Using a Choice-Format Survey. Value Heal. 2007;10(5):415–30.
