# Supplementary material for: Variation of colorectal, breast and prostate cancer screening activity in Switzerland: Influence of insurance, policy and guidelines
Source: PLoS One. 2020 Apr 16;15(4):e0231409. doi: 10.1371/journal.pone.0231409 (PMC7162274; doi:10.1371/journal.pone.0231409)
Supplement: S1 Fig — (PDF) [file pone.0231409.s004.pdf]

S4 Figure Swiss cantons with a breast cancer screening program in 2014

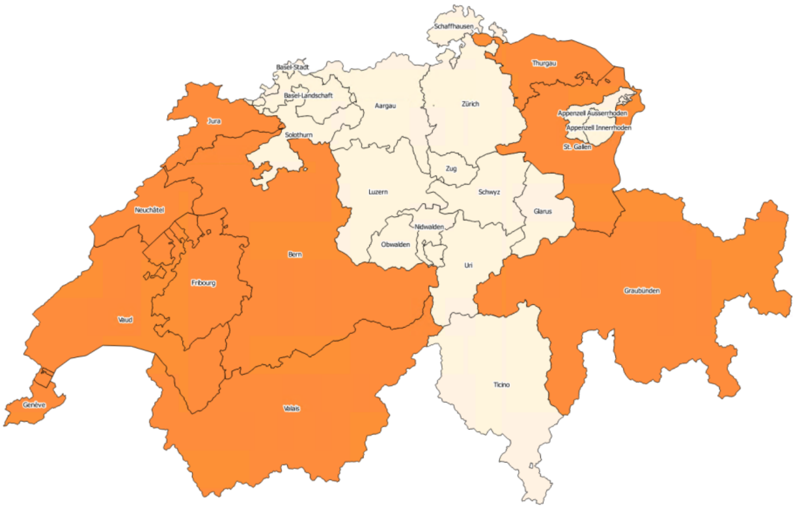

Note. Only programs that were present or introduced from the beginning of year 2014 are considered. The program in the canton of Basel city was started in the middle of 2014.
